# A Retrospective Analysis of the Effects of Pneumonia and Chronic Obstructive Pulmonary Disease on Patient Mortality in Southwest Missouri

**DOI:** 10.7759/cureus.76693

**Published:** 2024-12-31

**Authors:** Cameron Smith, Monika Ziogaite, Lindsay Doolan-Mattice, Heather VonHegel, Mariam Akhtar, Nova Beyersdorfer, Kerry Johnson, John Paulson

**Affiliations:** 1 Medicine, Kansas City University, Joplin, USA; 2 Primary Care, Kansas City University, Joplin, USA; 3 Family Medicine, Freeman Health System, Joplin, USA; 4 Mathematics Department, Missouri Southern State University, Joplin, USA

**Keywords:** copd: chronic obstructive pulmonary disease, copd mortality, lung disease, midwest, pneumonia

## Abstract

Chronic obstructive pulmonary disease (COPD) is a progressive lung disease. In this study, we aimed to explore the effects of COPD and pneumonia on the mortality rates among patients admitted to the Freeman Health System in Southwest Missouri, to provide a better idea of whether there is a correlation between the two and whether the presence of both adds to the mortality rates. Patient data were extracted from electronic medical records, focusing on patients with or without COPD and pneumonia diagnoses. Analyses revealed a statistical difference, with a p-value of <0.0001, in mortality rates between COPD patients with (P1) and without (P3) pneumonia, indicating that pneumonia did have an impact on COPD patients. However, pneumonia patients without COPD (P2) had a higher, but non-statistical difference in mortality rates compared to COPD patients with pneumonia (P1), with a p-value of 0.0806. These findings emphasize the severe influence of pneumonia in both COPD and non-COPD populations in the area, warranting further investigation and emphasizing the importance of timely and appropriate treatment. These results provide insights into the interplay between COPD and pneumonia in the geographic area, which may provide guidance in treatment decisions that may improve patient outcomes.

## Introduction

Chronic obstructive pulmonary disease (COPD) is a progressive and irreversible lung disease that affects over 14 million adults in the United States alone. It is currently the fourth leading cause of death in the United States, according to the National Institutes of Health [1]. COPD is the culmination of two main conditions, emphysema and chronic bronchitis. Both of these are often caused by long-term exposure to irritants, the most prevalent being cigarette smoke [2]. Emphysema is characterized by damage to the walls between the air sacs (alveoli) in the lungs and results in increased difficulty in exhalation [1]. The etiology of chronic bronchitis includes repeat or constant irritation and inflammation in the lining of the airways (bronchioles), which leads to the formation of thick mucus that can be challenging to remove through coughing, making it difficult to breathe [1]. In either of these cases, patients can present with symptoms that include, but are not limited to, coughing with or without phlegm, difficulty breathing, wheezing, and fatigue [3].

Patients with COPD are categorized into groups using the Global Initiative for Chronic Obstructive Lung Disease (GOLD) treatment algorithm, and then from there, the provider can determine appropriate initial pharmacological treatment as well as non-pharmacological approaches, if necessary [4]. The current standardized treatment for patients with stable COPD includes pharmacological therapies that aim to reduce symptoms and symptom severity, improve overall health status, and reduce the risk of exacerbations [4]. According to Yawn et al., some of these pharmacological therapies include an initial use of bronchodilators, which aim to increase airway diameter [5]. Depending on the severity of the disease and the patient’s symptoms, the patient could then potentially be placed on a long-acting muscarinic antagonist (LAMA) or a long-acting beta2-agonist (LABA). While inhaled corticosteroids (ICSs) are utilized for monotherapy in other obstructive lung diseases, such as asthma, they are not yet approved as a lone therapy for COPD patients [5]. This is due to evidence that shows substantial adverse effects from the use of ICSs in patients with COPD, most notably increased susceptibility to severe pneumonia leading to increased mortality [6].

An infection brought on by pathogens, like viruses or bacteria, resulting in the inflammation of alveoli in the lungs, is called pneumonia, which results in symptoms including cough, fever, and difficulty breathing [2]. When an individual has pneumonia, the alveoli in the lungs are filled with pus and fluid. This fluid limits gas exchange and can not only be extremely painful but can cause life-threatening respiratory issues as well [3]. Understanding the interplay between COPD and pneumonia is essential for medical professionals as it can guide treatment decisions and significantly impact patient outcomes. According to a study conducted in 2018, COPD is associated with a higher risk of developing more severe pneumonia compared to those without COPD. In addition, this study found that patients with pneumonia and a diagnosis of COPD showed a significantly higher 30- and 90-day mortality rate, than non-COPD patients [7].

Our study was conducted to further investigate the relationship between pneumonia and COPD, and whether the presence of both diseases would cause a significant increase in mortality rates. Knowing that pneumonia is a deadly disease, we focused on comparing mortality rates of patients who had pneumonia without a diagnosis of COPD to patients with pneumonia and a corresponding diagnosis of COPD. Our study findings can potentially provide additional information to further research and substantiate whether or not pneumonia is adding to mortality rates.

## Materials and methods

Data collection

Data were obtained from the Freeman Health System in Joplin and Neosho, Missouri, and extracted from electronic medical records (EMR) using the International Classification of Diseases, 10th Revision (ICD-10) codes (Tables 1-2) [8]. The study included patients discharged between January 1, 2019, and December 31, 2021, and only patients aged 18 and above were included in the analysis. The criteria for pneumonia diagnosis based on ICD-10 codes (listed in Table 1) were nonspecific and only required a diagnosis without prior admission for pneumonia to be considered an inclusion diagnosis. Patients were then reclassified based on their prior COPD diagnosis to analyze the data further, using specific ICD-10 codes as inclusion criteria (listed in Table 2). Patients with any prior admission were excluded from the data. Patient data were collected if patients were discharged from the hospital during the specified timeframe and met the inclusion criteria based on ICD-10 codes for pneumonia and COPD (Tables 1-2).

**Table 1:** International Classification of Diseases, 10th Revision (ICD-10) codes for pneumonia

ICD-10 codes	Diagnosis
J1000	Influenza due to other identified influenza virus with unspecified type of pneumonia
J1001	Influenza due to other identified influenza virus with the same other identified influenza virus pneumonia
J1008	Influenza due to other identified influenza virus with other specified pneumonia
J1100	Influenza due to unidentified influenza virus with unspecified type of pneumonia
J1108	Influenza due to unidentified influenza virus with specified pneumonia
J120	Adenoviral pneumonia
J121	Respiratory syncytial virus pneumonia
J122	Parainfluenza virus pneumonia
J123	Human metapneumovirus pneumonia
J1281	Pneumonia due to SARS-associated coronavirus
J1282	Pneumonia due to coronavirus disease 2019
J1289	Other viral pneumonia
J129	Viral pneumonia, unspecified
J13	Pneumonia due to Streptococcus pneumoniae
J14	Pneumonia due to Hemophilus influenzae
J150	Pneumonia due to Klebsiella pneumoniae
J151	Pneumonia due to Pseudomonas
J1520	Pneumonia due to staphylococcus, unspecified
J15211	Pneumonia due to methicillin-susceptible Staphylococcus aureus
J15212	Pneumonia due to methicillin-resistant Staphylococcus aureus
J1529	Pneumonia due to other staphylococcus
J153	Pneumonia due to streptococcus, group B
J154	Pneumonia due to other streptococci
J155	Pneumonia due to Escherichia coli
J156	Pneumonia due to other Gram-negative bacteria
J157	Pneumonia due to Mycoplasma pneumoniae
J158	Pneumonia due to other specified bacteria
J159	Unspecified bacterial pneumonia
J168	Pneumonia due to other specified infectious organisms
J17	Pneumonia in diseases classified elsewhere
J180	Bronchopneumonia, unspecified organism
J181	Lobar pneumonia, unspecified organism
J188	Other pneumonia, unspecified organism
J189	Pneumonia, unspecified organism
J84116	Cryptogenic organizing pneumonia
J851	Abscess of lung with pneumonia
J95851	Ventilator-associated pneumonia

**Table 2:** International Classification of Diseases, 10th Revision (ICD-10) codes for chronic obstructive pulmonary disease

ICD-10 codes	Diagnosis
J440	Chronic obstructive pulmonary disease with (acute) lower respiratory infection
J441	Chronic obstructive pulmonary disease with (acute) exacerbation
J449	Chronic obstructive pulmonary disease, unspecified

The data used in this study reflect patients from the geographic area encompassing Oklahoma, Arkansas, Kansas, and Missouri, and included information on pneumonia diagnosis, COPD diagnosis, and mortality outcomes.

Data analysis

The collected data were analyzed to compare the outcomes between patients with and without pneumonia and to explore any potential influence of a prior diagnosis of COPD on pneumonia outcomes. The main outcome of interest was the mortality rate. Patients were classified into different sample populations based on their diagnosis of pneumonia and COPD. Data for three populations were extracted for this study: patients who were diagnosed with pneumonia and had COPD were classified as P1, patients with pneumonia but without COPD as P2, and patients with COPD without pneumonia as P3.

Statistical analyses, including descriptive statistics and hypothesis testing, were conducted to determine any significant differences in mortality rates among the sample populations. Confidence intervals (CIs) were calculated to assess the precision of the estimates along with p-values to assess significance.

A total of 5,128 patients diagnosed with pneumonia and admitted to the hospital were identified. Among them, 714 prior admissions were excluded from the study. The remaining 4,414 patients in the pneumonia group were then divided into groups based on the diagnosis of COPD. From this group, 1,730 patients were selected for the group with both pneumonia and COPD, which became the P1 sample population. Additionally, 2,684 patients were selected for the group with pneumonia but no COPD, becoming the P2 sample population.

Furthermore, 31,562 patients diagnosed without pneumonia and admitted to the hospital were identified. Initially, 4,052 patients were excluded due to a prior diagnosis of pneumonia. The remaining 27,510 patients were then divided into groups using COPD as inclusion criterion. At this stage, 1,997 prior admissions were excluded, and 21,177 patients were excluded due to the absence of a COPD diagnosis. The remaining 4,336 patients with no pneumonia but had COPD represented the P3 sample population.

The study aimed to compare mortality rates in the sample populations and statistically analyze these rates for any significance. Mortality rates were determined based on the number of patients who died during their hospital stay. Statistical analyses, including descriptive statistics and hypothesis testing, were conducted to determine any significant differences in mortality rates among the sample populations. Mortality rates for each sample population were then used to find the sample proportion using Wald's method [9]. CIs were computed using a two-sample proportion summary hypothesis test to assess the statistical significance of the mortality rates. Any significant differences in mortality rates could be identified by comparing the CIs between different sample populations.

Ethical considerations

This study was conducted in accordance with ethical guidelines and regulations. As a retrospective study using de-identified data, informed consent was not required. Patient confidentiality and privacy were maintained throughout the data collection and analysis process. Freeman Health System’s Institutional Review Board reviewed and approved the study protocol (approval no. 2022003).

## Results

**Figure 1:**
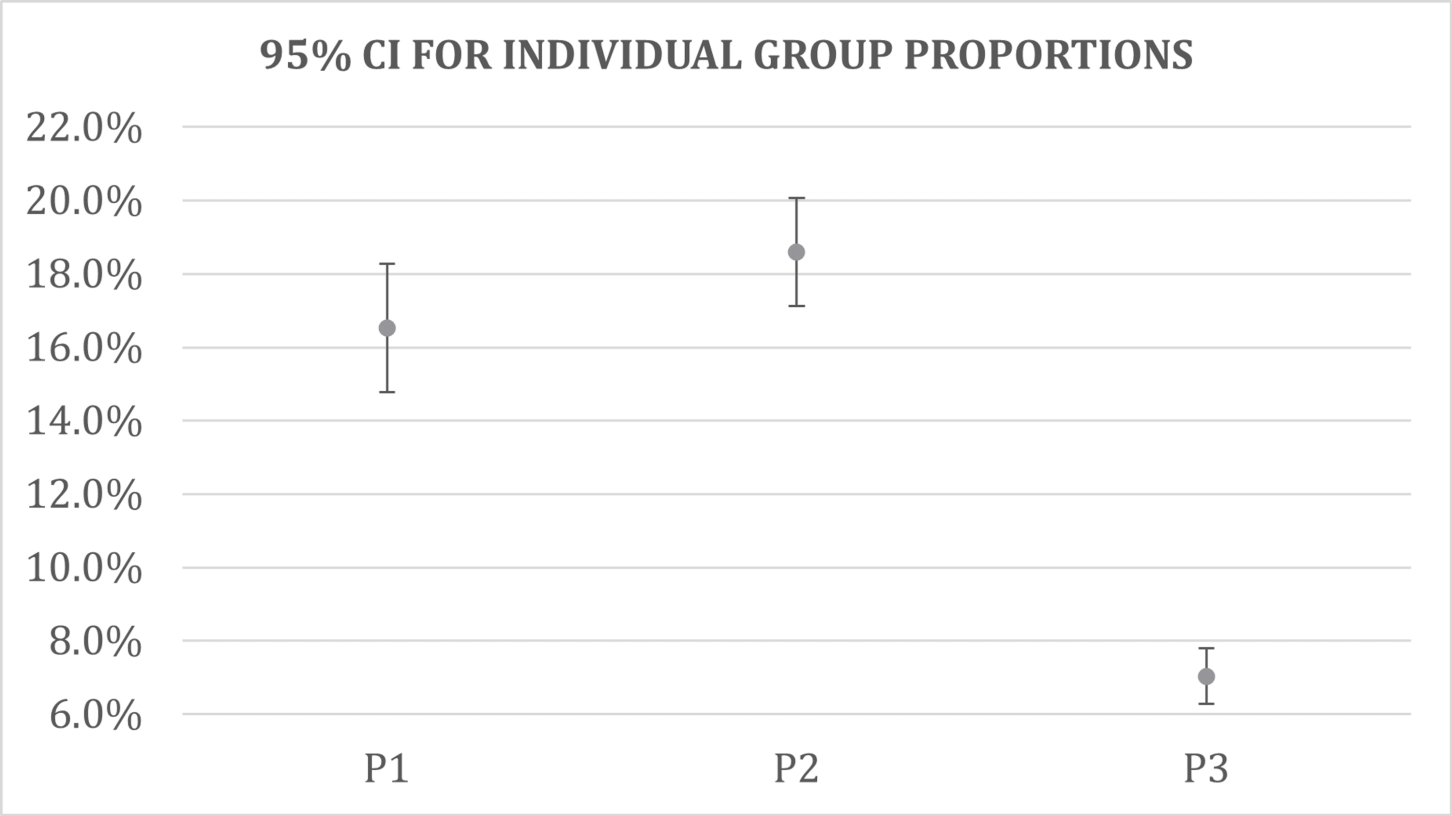
Individual group proportion test P1: group with COPD and pneumonia; P2: group with pneumonia, without COPD; P3: group with COPD, without pneumonia

Of the 1,730 patients with both COPD and pneumonia (P1), 286 died in the hospital (16.53%). A total of 499 of the 2,684 evaluated with pneumonia and without COPD (P2) also died in the hospital (18.59%). Among the patients with COPD and without pneumonia (P3), 305 out of 4,336 patients died while in the hospital (7.03%); the remainder of all groups were otherwise discharged from the facility. Individual group data for mortality are shown in Figure 1. Interestingly, sample mortality rates were found to be the highest in patients with pneumonia and without COPD as compared to the two COPD groups. As shown in Table 3, mortality was 16.53% for P1, 95% CI (14.78%, 18.28%); 18.59% for P2, 95% CI (17.12%, 20.06%) and 7.03% for P3, 95% CI (6.27%, 7.80%).

**Table 3:** Individual mortality data P1: group with COPD and pneumonia; P2: group with pneumonia, without COPD; P3: group with COPD, without pneumonia

Sample populations	Mortality (n)	Sample proportion	Lower 95% CI	Upper 95% CI
P1	286 of 1,730	16.53%	14.78%	18.28%
P2	499 of 2,684	18.59%	17.12%	20.06%
P3	305 of 4,336	7.03%	6.27%	7.80%

**Figure 2:**
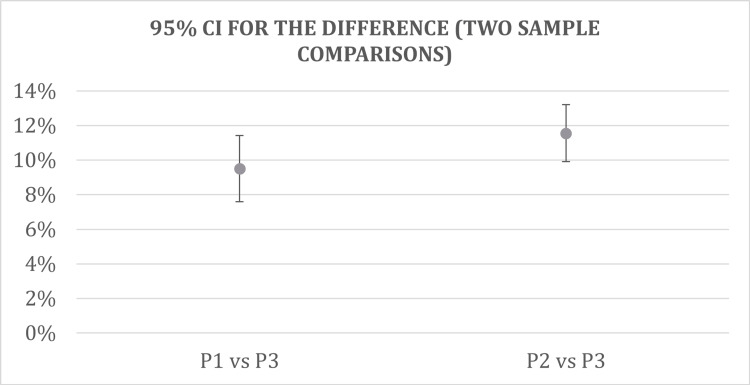
Two-sample proportion test results P1: group with COPD and pneumonia; P2: group with pneumonia, without COPD; P3: group with COPD, without pneumonia

Analysis results of two-sample proportion tests are shown in Table 4; p < 0.05 was used to determine significance. On comparing sample mortality rates of patients in groups P1, P2, and P3 (Figure 2), mortality was found to be 2.06% higher for P2 vs. P1, which was found to be an insignificant difference. There was an 11.56% higher sample mortality rate for P2 when compared to P3, 95% CI (9.90%, 13.21%). Data suggested that patients with pneumonia had statistically significant higher sample mortality rates as compared to patients with either COPD without pneumonia or COPD and pneumonia, potentially indicating pneumonia to be the deadlier disease. Figure 2 delineates this trend, showing patients with pneumonia to have a significantly higher sample mortality rate as compared to those without pneumonia.

**Table 4:** Mortality data comparisons P1: group with COPD and pneumonia; P2: group with pneumonia, without COPD; P3: group with COPD, without pneumonia

Comparison	Mortality Sample 1	Mortality Sample 2	Difference in mortality rates	Lower 95% CI for P1-P2	Upper 95% CI for P1-P2	p-value
P1 vs. P2	286 of 1,730	499 of 2,684	0.0206	-	-	0.0806
	0.1653	0.1859				
P1 vs. P3	286 of 1,730	305 of 4,336	0.0950	0.0759	0.1141	<0.0001
	0.1653	0.0703				
P2 vs. P3	499 of 2,684	305 of 4,336	0.1156	0.0990	0.1321	<0.0001
	0.1859	0.0703				

## Discussion

This study aimed to determine if the presence or absence of COPD had a significant impact on mortality in patients with pneumonia. From 2019 to 2021, in-patient data were collected from a hospital system in Southwest Missouri and analyzed. The results not only answered the proposed question but also gave two other conclusions regarding COPD and pneumonia. As can be deduced from Table 4, there was no significant difference in patients with both COPD and pneumonia (P1) when compared to patients with pneumonia but without COPD (P2) (p = 0.0806), which suggests that COPD did not affect mortality rates in pneumonia patients. Secondly, there was a significant difference between patients with COPD but without pneumonia (P3) and P1 patients (p < 0.0001), indicating that pneumonia did affect mortality rates in patients with COPD. Moreover, there was a significant difference between P2 and P3 (p < 0.0001), leading to the conclusion that pneumonia is a deadlier disease than COPD when both are compared individually.

The question about whether COPD has an effect on mortality in patients with pneumonia was answered by comparing P1 and P2. When comparing these groups, it was found that there was not a statistically significant difference between P1 and P2, indicating that COPD neither increases nor decreases the mortality rate in patients with pneumonia. Although this finding seems odd at face value, considering one would think that a comorbidity as serious as COPD would likely increase the mortality rate in patients with pneumonia, there has been research in the past that agrees with this conclusion [10]. Studies in the past concluded that it could be due to ICSs which patients are often prescribed when battling COPD [11-13]. ICSs have been seen to lead to an increased incidence of pneumonia but there appears to be no change or decrease in mortality in COPD patients taking ICSs [10,14,15].

Results for P1 vs. P3 and P2 vs. P3 showed that any group with pneumonia had a higher mortality rate than groups with COPD. When comparing P1 vs. P3, patients with pneumonia and COPD had a significantly higher mortality rate than patients without COPD. When examining P2 vs. P3, we saw that patients with pneumonia had a higher mortality rate than patients with COPD, without pneumonia. The results of these comparisons, that pneumonia has a higher mortality rate than COPD, can be explained by several considerations. Pneumonia is an acute disease process when compared to the chronic disease process of COPD; thus, patients severely ill with pneumonia are more likely to be admitted and be transferred to in-patient facilities compared to patients with chronic symptoms of COPD who may be more likely to be discharged with follow-up. Essentially, while it is true that COPD is a fatal disease with a high mortality rate, our observations of admitted patients show that there is a higher mortality rate in patients with pneumonia when compared to COPD [7]. Since these data were collected only for patients admitted to the hospital, these patients were more likely to die from an acute illness such as pneumonia rather than a chronic illness like COPD [15]. Also, patients with a history of COPD may be reluctant to seek medical help while sick as the years of illness can take a toll on their mental and physical health while also mistaking their pneumonia for a bout of COPD exacerbation; these patients may be less likely to seek help until it is too late, leading to a patient dying prior to the patient’s arrival at the hospital or in the emergency department [16,17].

It is important to note that during the study period from 2019 to 2021, hospitals experienced higher rates of pneumonia due to the COVID-19 pandemic [18,19]. This increase in pneumonia cases could have influenced the study results as patients with COVID-19-induced pneumonia are known to have higher mortality rates compared to pneumonia not associated with COVID-19 [20,21].

Limitations

Due to the non-random sampling of the data, the results cannot be assumed to be representative of a population as a whole; in addition, as with all retrospective studies, this study cannot prove causality. This study used ICD-10 codes to determine the presence of COPD and/or pneumonia and thus there is a possibility for errors, including incorrect inclusion of subjects without the diseases noted or exclusion of subjects who did not have the appropriate diagnosis placed into the electronic medical records during their care. The data did not account for age, gender, severity or stage of disease, additional comorbidities, or other confounding variables between groups. These additional variables could have played a role in the outcomes of these patients and the results of the study.

## Conclusions

In this study, no significant difference was found in mortality rates for pneumonia patients with or without COPD. Further research needs to be done to explore this finding, as well as the possible connection between these findings and ICSs. In addition, our study found that pneumonia is associated with a higher mortality rate in admitted patients when compared to COPD.
